# Editorial: Application of computational intelligence techniques for lifestyle related diseases management

**DOI:** 10.3389/fdgth.2026.1832287

**Published:** 2026-05-01

**Authors:** H. L. Gururaj, Hong Lin, Francesco Flammini, J. Shreyas

**Affiliations:** 1 Manipal Institute of Technology Bengaluru, Manipal Academy of Higher Education, Manipal, India; 2 University of Houston, Houston, TX, United States; 3 University of Florence - Department of Mathematics and Computer Science “Ulisse Dini” (DIMAI), Florence, Italy; 4 University of Applied Sciences and Arts of Southern Switzerland - Dalle Molle Institute for Artificial Intelligence (IDSIA USI-SUPSI), Lugano, Switzerland

**Keywords:** blood glucose, lifestyle recommendation, patient, random forest, XGBoost

The global burden of chronic illnesses continues to rise, leading to a growing population living with multimorbidity (the co-occurrence of two or more chronic conditions). Lifestyle-related non-communicable diseases (NCDs)—including diabetes and cardiovascular disease—contribute substantially to morbidity and mortality worldwide, and their effective management increasingly depends on continuous monitoring, timely decision-making, and sustained behavior change. For diabetes in particular, conventional glucose monitoring methods can be invasive, uncomfortable, and anxiety-provoking for patients; therefore, non-invasive alternatives are being actively explored but remain constrained by cost and accuracy considerations.

Healthy lifestyle behaviors have been associated with improved prognosis and better quality of life among breast cancer survivors. However, maintaining lifestyle changes over time is challenging, highlighting the need to identify the most effective components of lifestyle education programs and to tailor interventions that improve adherence and facilitate integration into routine care.

This Special Issue showcases recent advances in applying computational intelligence (CI)—including machine learning, deep learning, and data-driven decision support—to the prevention, monitoring, and management of lifestyle-related diseases. In line with its objectives, the contributions span scalable digital self-health management for multimorbidity, non-invasive sensing and forecasting for diabetes management, and predictive modeling of lifestyle adherence and quality-of-life outcomes.

Wang et al., in the paper titled “*Effects of Internet Self-Health Management on Patients with Chronic Disease Multimorbidity: A 4-Year Longitudinal Study*”, evaluated the effectiveness of internet-based self-health management in improving health behaviors and clinical indicators in patients with multimorbidity.

A total of 30,745 adults aged ≥18 years were recruited across five provinces in northwestern China. Following baseline data collection in 2013, participants received structured internet-based health guidance covering diet and nutrition, exercise, and mental health. In 2017, follow-up questionnaires and clinical measurements were used to assess changes in health behaviors and clinical indicators among 2,535 participants with multimorbidity. Binary logistic regression models were applied to identify factors associated with multimorbidity management outcomes.

Khan et al., in their article “*Non Invasive Blood Glucose Estimation using Green Light Photoplethysmography and Machine Learning*”, investigated the potential of using green light photoplethysmography (PPG) signals for non-invasive blood glucose monitoring.

A custom-designed PPG acquisition system was developed to record signals from 80 subjects under controlled conditions. Capillary blood glucose was measured using a lancing device as the reference standard. Model performance was evaluated using the coefficient of determination (R^2^), mean absolute error, and bias analysis.

Among the tested models, random forest regression (RFR) showed the best performance with an R^2^ value of 0.92 and a mean absolute error of 4.8 mg/dl. Predicted glucose levels demonstrated minimal bias across glucose ranges, with mean differences of −5.11 ± 0.80 mg/dl (<90 mg/dl), −3.68 ± 1.24 mg/dl (90–120 mg/dl), and −5.61 ± 0.75 mg/dl (≥120 mg/dl). These findings suggest that PPG signals in the green-light spectrum can effectively reflect blood glucose levels, supporting their potential for non-invasive glucose monitoring.

In the article “*An AI-Based Module for Interstitial Glucose Forecasting enabling a “Do-It-Yourself” Application for People with Type 1 Diabetes*”, Rodriguez-Almeida et al. proposed a deep-learning-based do-it-yourself (DIY) framework for interstitial glucose prediction using continuous glucose monitoring (CGM) data, with one personalized model trained per user (without relying on data from other individuals).

The DIY module reads CGM raw data (as it would be uploaded by potential users) and automatically prepares the dataset to train and validate a deep learning (DL) model for glucose prediction up to one hour ahead. For training and validation, one year of CGM data from 29 individuals with type 1 diabetes (T1D) was used.

Results showed prediction performance comparable to the state of the art while using only CGM data. To the best of our knowledge, this work is the first to provide a DL-based DIY approach for fully personalized glucose prediction. Moreover, the framework is open source and has been deployed in Docker, enabling standalone use, integration into a smartphone application, and experimentation with novel DL architectures.

In the article “*Adopting machine learning to predict breast cancer patients adherence with lifestyle recommendations and quality of life outcomes*”, Crispo et al. analyzed data from 316 breast cancer survivors.

Adherence was modeled as a multi-label time-series classification task, with compliance recorded on a three-point scale for each treatment component at quarterly intervals over one year. Health-related quality of life (HRQoL) was predicted using first-year adherence data to estimate the mean score over the subsequent three measurements.

An XGBoost regressor was used for HRQoL prediction and was compared to a baseline linear regression model. XGBoost demonstrated superior predictive performance, achieving an R-squared value of 0.62.

Overall, the articles in this Special Issue demonstrate how computational intelligence can complement lifestyle medicine by enabling scalable self-management interventions, less burdensome monitoring, and personalized prediction to support timely decisions. Future work should prioritize external validation across diverse populations, transparent and interpretable models, and secure integration with clinical workflows and wearable/mobile platforms. We hope this collection stimulates further research and translation of CI-driven solutions that improve outcomes and quality of life for people living with lifestyle-related diseases. We thank the authors, reviewers, and the Frontiers editorial staff for their contributions and support.

